# Adipocyte-Derived Leptin Promotes PAI-1-Mediated Breast Cancer Metastasis in a STAT3/miR-34a Dependent Manner

**DOI:** 10.3390/cancers12123864

**Published:** 2020-12-21

**Authors:** Si-Jing Li, Xiao-Hui Wei, Xiao-Man Zhan, Jin-Yong He, Yu-Qi Zeng, Xue-Mei Tian, Sheng-Tao Yuan, Li Sun

**Affiliations:** 1 New Drug Screening Center, China Pharmaceutical University, Nanjing 210009, China; lsj906200413@163.com (S.-J.L.); 1821071005@stu.cpu.edu.cn (X.-M.Z.); hejy68@mail2.sysu.edu.cn (J.-Y.H.); 1822081088@stu.cpu.edu.cn (Y.-Q.Z.); 1621010264@cpu.edu.cn (X.-M.T.); 2 School of Basic Medical Sciences, Anhui Medical University, Hefei 230032, China; weixiaohui@ahmu.edu.cn; 3 China Cell-gene Therapy Translational Medicine Research Center, Biotherapy Center, The Third Affiliated Hospital of Sun Yat-sen University, Guangzhou 510630, China; 4 School of Medicine, Sun Yat-sen University, Shenzhen 518107, China; 5 Jiangsu Center for Pharmacodynamics Research and Evaluation, China Pharmaceutical University, Nanjing 210009, China

**Keywords:** adipocytes, breast cancer metastasis, leptin/OBR, STAT3, miR-34a, PAI-1

## Abstract

**Simple Summary:**

Although adipocytes affect the metastatic behavior of cancer cells, the underlying molecular mechanisms remain largely elusive. Thereby, we sought to screen for the signaling pathways responsible for adipocyte-induced motility of breast cancer cells by employing a breast cancer cell/adipocyte coculture system. Our study revealed that adipocyte coculture stimulated PAI-1 expression in breast cancer cells to potentiate cell motility. Furthermore, we obtained evidence that adipocytes secreted leptin to activate OBR in breast cancer cells, which phosphorylated STAT3 to promote the transcription of PAI-1 and repress the expression of miR-34a as the negative regulator of PAI-1. Our study provides new evidence for the involvement of adipocytes in breast cancer evolution, which advances the evolving roles of stromal cells in tumor pathogenesis.

**Abstract:**

The crosstalk between cancer cells and adipocytes is critical for breast cancer progression. However, the molecular mechanisms underlying these interactions have not been fully characterized. In the present study, plasminogen activator inhibitor-1 (PAI-1) was found to be a critical effector of the metastatic behavior of breast cancer cells upon adipocyte coculture. Loss-of-function studies indicated that silencing PAI-1 suppressed cancer cell migration. Furthermore, we found that PAI-1 was closely related to the epithelial-mesenchymal transition (EMT) process in breast cancer patients. A loss-of-function study and a mammary orthotopic implantation metastasis model showed that PAI-1 promoted breast cancer metastasis by affecting the EMT process. In addition, we revealed that leptin/OBR mediated the regulation of PAI-1 through the interactions between adipocytes and breast cancer cells. Mechanistically, we elucidated that leptin/OBR further activated STAT3 to promote PAI-1 expression via miR-34a–dependent and miR-34a–independent mechanisms in breast cancer cells. In conclusion, our study suggests that targeting PAI-1 and interfering with its upstream regulators may benefit breast cancer patients.

## 1. Introduction

Metastasis remains hard to control and it is the greatest contributor to deaths from breast cancer [1]. Although cancer cells represent the main driving force of metastasis, the tumor microenvironment (TME) is indispensable in guiding metastatic behavior [2,3]. As cancer progresses, tumor cells interact closely with stromal cells by cytokine-mediated communication or direct cell-cell contact, which results in multiple signaling pathway crosstalk to potentiate the aggressiveness and metastasis of cancer cells [4]. The stromal cells in the breast cancer TME include cancer-associated fibroblasts (CAFs), mesenchymal stem cells, adipocytes, endothelial cells, pericytes and immune cells. Among these cells, the roles of CAFs and tumor-associated macrophages (TAMs) in breast cancer metastasis have been extensively investigated, and the focus has been on secreted factor-mediated interaction such as growth factors, inflammatory factors, integrins, proteases and chemokines [5,6,7,8,9,10,11,12,13]. Recently, interest has extended to other cell types in the breast cancer TME, such as adipose stem cells (ASCs), cancer-associated adipocytes (CAAs) and adipocytes [14,15,16]. 

Adipocytes are the major cell type in the mammary fat pad, therefore, it is reasonable to focus on adipocytes in breast cancer metastasis. To date, extracellular matrix (ECM) remodeling induced by proteases, matrix proteins, or CAF-like phenotypic changes has been actively studied in the adipocyte-driven metastatic behavior of breast cancer cells [15,17,18]. In addition, the driving force of adipocytes in breast cancer metastasis can also be attributed to cytokine-mediated communication between these cells, which results in the upregulation of metastasis-related factors such as PLOD2 and MMP-2 in cancer cells [14,16,19,20]. Although adipokine-mediated crosstalk, such as IL-6, leptin, resistin, and autotaxin, has been highlighted in adipocyte-cancer cell interactions [14,16,19,20], the detailed mechanisms are not fully defined. In addition, the clinical correlation of such crosstalk in breast cancer remains largely unclear. 

Plasminogen activator inhibitor-1 (PAI-1/SERPINE1) is a primary member of the serpin superfamily, and functions as an inhibitor of tissue plasminogen activator (tPA) and urokinase-type plasminogen activator (uPA) [21,22,23,24,25,26]. PAI-1 is essential in thrombosis. In addition, an increase in the PAI-1 level is closely related to various pathological states, such as metabolic syndrome, vascular diseases and various cancer types [21]. Indeed, higher PAI-1 levels have been detected in multiple types of cancer biopsy tissues or plasma than in normal controls [22,23,24]. More importantly, PAI-1 has been considered an indicator to evaluate cancer progression and relapse in certain cancer types [22,23,24]. PAI-1 mainly functions as a promoter in cancer development, as characterized by inducing tumor vascularization, cell proliferation, tumor growth, tumor metastatic behavior, etc. [25,26]. Our group previously detected high levels of PAI-1 in the conditioned medium (CM) of breast cancer cells upon adipocyte coculture. We also described PAI-1 as an inducer of ECM remodeling that contributed to tumor metastasis [15]. Nonetheless, whether PAI-1 mediates adipocyte-driven metastatic behavior of breast cancer cells remains unclear. In addition, upstream regulators of PAI-1 and the underlying mechanisms are incompletely understood.

MicroRNAs (miRNAs) are a family of small non-coding RNAs, which have been implicated in the regulation of tumor development. Accumulating evidence showed that miRNAs can serve as tumor suppressors by binding with specific messenger RNAs (mRNAs) to induce translational repression or mRNA degradation [27]. As a member of miRNAs, miR-34a was lower expressed in aggressive breast cancer cell lines compared with less aggressive cell lines and frequently downregulated in breast cancer tissues compared with normal tissues [28,29]. In addition, low expression of miR-34a predicted poor outcome in breast cancer patients, which was verified by clinical data, a systematic review, and huge number of cases meta-analysis [30]. Functionally, miR-34a attenuated breast cancer progression by targeting cancer-related genes such as Fra-1, FOXM1, eEF2K, and NOTCH1, leading to reduced tumor growth, metastasis, stemness, and chemoresistance [28,29,31,32]. Although miR-34a repressed the metastasis of non-small-cell lung cancer (NSCLC) by targeting PAI-1 [33], the regulation of PAI-1 by miR-34a in breast cancer remains unclear.

In this study, PAI-1 was validated as a key driver accounting for adipocyte-driven metastasis of breast cancer cells. In addition, clinical data suggested that PAI-1 was closely related to mesenchymal features in breast cancer. From a clinical perspective and based on the adipokine-receptor principle, leptin/OBR was found to be closely associated with PAI-1 expression in breast cancer. Mechanistically, we revealed that adipocyte-derived leptin activated STAT3 to directly enhance PAI-1 expression, or indirectly affect the PAI-1 level by repressing miR-34a, thereby promoting PAI-1-induced breast cancer metastasis.

## 2. Materials and Methods 

### 2.1. Cell Culture and Reagents

Breast cancer cell lines (MDA-MB-231, SKBR-3, and MCF-7) were obtained from the Shanghai Institute of Life Science, Chinese Academy of Sciences (Shanghai, China) and maintained in DMEM (Thermo Fisher Scientific, Waltham, MA, USA) containing 10% FBS (PAN-Biotech, Adenbach, Bavaria, Germany), 50 units/mL penicillin and 50 units/mL streptomycin. All these cells have been authenticated by short tandem repeats analysis (Genetic Testing Biotechnology Corporation, Suzhou, China). The 3T3-L1 preadipocyte cell line was obtained from American Type Culture Collection (Manassas, VA, USA) and the growth and differentiation of these cells were performed as previously described [15]. Cells were cultivated in DMEM containing 10% fetal calf serum (FCS) (Thermo Fisher Scientific) and reached confluency after 3 days. For further differentiation, these cells were maintained in DMEM supplemented with 10% FBS (Gibco, Thermo Fisher Scientific), 0.5 mM IBMX (Sigma, St. Louis, MO, USA), 1 μM dexamethasone (Sigma), and 10 μg/mL insulin (Sigma) for 3 days, subsequently, cultured in DMEM containing 10% FBS and 10 μg/mL insulin for 6 days until they reached full differentiation, and then exposed to DMEM containing 10% FBS for 3 days. oil red O staining and BODIPY staining were performed to confirm differentiation efficiency. 

### 2.2. Coculture, Conditioned Medium Collection and Migration Assays

For coculture assays, 2 × 10^5^–3 × 10^5^ breast cancer cells (MDA-MB-231, SKBR-3, MCF-7) were seeded into transwell inserts (0.4 μm pore size membrane, Corning, Corning, NY, USA) for 12 h. Before cancer cells were suspended above the wells of differentiated adipocytes, the medium in upper or down wells was replaced with serum-free DMEM and then cocultured for 72 h, 48 h, 24 h, 12 h, 6 h, and 0 h. The conditioned medium and breast cancer cells at the indicated time were collected for protein extraction and western blot analysis, while those collected at 0 h (monoculture) were considered as controls.

To analyze the effect of conditioned medium on certain protein expression in breast cancer cells, the conditioned medium in the upper transwell inserts and lower wells after coculture for 72 h and 0 h were separately collected and centrifuged for 5 min at 1000 rpm. Then 1mL conditioned medium supplemented with 2% FBS was added to already adherent breast cancer cells in 12-well plates for 72 h, followed by collecting cells for western blot analysis.

To analyze migration, 5 × 10^4^ MDA-MB-231 or 2 × 10^5^ SKBR-3 cells, previously cocultured with adipocytes for 72 h, were seed into the upper transwell inserts (8.0 μm pore size membrane, Corning) in serum-free medium, while 10% FBS added to the lower wells as a chemoattractant. After 12 h or 24 h, the cells at the bottom of the transwell inserts were fixed and stained with Diff-Quick stain kit (Jiancheng, Nanjing, China), and the images of five fields per membrane were taken and counted using Image J (NIH, Bethesda, MD, USA) https://imagej.nih.gov/ij/docs/intro.html.

### 2.3. Wound Healing Scratch Assays and 3D Invasion Assays

After coculture with adipocytes for 72 h, breast cancer cells (MDA-MB-231, SKBR-3) were collected and seeded into 12-well plates for 24 h until reaching 90% confluence. Then scratches were generated by using a yellow pipette tip and cultured in serum-free medium for 48 h after washing three times with medium. Images were captured by an inverted microscope (Olympus, Tokyo, Japan) at 0 h, 24 h, and 48 h and the scratches were analyzed by Image J (NIH).

For 3D invasion assays, the hanging-drop method was used to generate 3D spheroids [34]. Breast cancer cells (MDA-MB-231, SKBR-3), cultured at the presence or absence of adipocytes for 72 h, were collected and re-suspended in DMEM containing 30% methylcellulose (Sigma) and 5% growth factor-reduced Matrigel (BD Biosciences, Franklin Lakes, New Jersey, USA), followed by incubating as droplets (30 μL) containing 3.5 × 10^4^ for 24 h to generate spheroids. To analyze invasion, 96-well plates were firstly coated with 50 μL Matrigel (BD Biosciences) for each well and added 200 μL medium before the spheroids were moved to the wells by yellow pipette tip. The medium was refreshed every 3 days and images were captured by using inverted microscope (Olympus, Tokyo, Japan) at day 0, 3, and 7 respectively.

### 2.4. Cytokine Protein Arrays

Adipocytes and cancer cells were cocultured or cultured alone for 72 h followed by the collection of conditioned medium (CM). Then the concentration of cytokines in CM was detected by using a cytokine array kit (RayBiotech, Norcross, GA, USA) based on the manufacturer’s instructions. 

### 2.5. Transfection and Generation of Stable Cells

MDA-MB-231 cells stably silencing PAI-1 (Sh1, Sh2) were generated as previously described [15]. To establish MDA-MB-231 cells stably expressing LEPR/OBR (MDA-MB-231/OBR), Full-length cDNA of a lentiviral-carrying human LEPR with GFP (pLenti-CMV-GFP-Puro, detailed information is shown in File S1) was purchased from PPL (Nanjing, China). LEPR or negative control plasmids (FF2) together with pMD2.G and psPAX2 plasmids were transfected to 293T cells with FuGENE^®^ 6 (Promega, Madison, WI, USA) for 48 h, followed by refreshing the medium and incubating for 48 h to generate lentiviruses. Then MDA-MB-231 cells were incubated with above conditioned medium for 72 h and selected with puromycin (10 μg/mL) to generate cells stably expressing LEPR.

### 2.6. Transient Transfection with siRNA or miR-34a Mimic

SERPINE1, LEPR, or STAT3 were knocked down in breast cancer cells by using SERPINE1-, LEPR- or STAT3-specific siRNAs, and a non-targeting siRNA sequence (NC) was used as the control, which were all obtained from GenePharma (Shanghai, China). miR-34a mimic and negative control (NC) were purchased from RiboBio (Guangzhou, China). Breast cancer cells were seeded at 50% confluence into six-well plates 12 h before the transfection, then siRNAs, miR-34a mimic, and corresponding NC were diluted in serum-free medium and transfected using Lipofectamine 2000 (Thermo Fisher Scientific) according to the manufacturer’s instructions. After 8 h, the medium was refreshed with a complete medium and incubated for 48–72 h, then the cells were harvested for qRT-PCR, western blotting, co-culture, or transwell migration assays. For coculture assays, the medium was supplemented with 2% FBS. Bulge-Loop miRNA qRT-PCR Starter Kit obtained from RiboBio was used to detect miR-34a expression and U6 was used as the internal control. The RT primers, forward and reverse primers of miR-34a and U6 were all purchased from RiboBio. The siRNA of PAI-1, OBR, and STAT3, and paired NC sequences are listed in Table S1.

### 2.7. Recombinant Human Leptin 

2 × 10^5^–3 × 10^5^ breast cancer cells were seed into 6-well plates for 12 h followed by external leptin (GeneScript, Piscataway, NJ, USA) treatment with concentration ranging from 25 ng/mL to 400 ng/mL or for 0 h to 72 h. Then samples were collected at indicated time points for further western blot or qRT-PCR analysis. miR-34a mimic or STAT3 siRNA were transfected into breast cancer cells for indicated time, then exposed to leptin for 72 h followed by qRT-PCR or Immunofluorescence.

### 2.8. In Vivo Studies

All animal experimental protocols were approved by the Institutional Animal Care Committee of China Pharmaceutical University. Mammary adipose tissues from breast cancer patients (>2 cm away from tumors) were provided by Zhong-Da Hospital of Southeast University less than 1 h after surgery. The study was performed using five to six-week old NOD/SCID mice (Nanjing University, Nanjing, China) with six used per group, and each mouse was subcutaneously implanted 1 mL of human adipose tissue to form a fat pad. After 1 week, MDA-MB-231 cells labeled with GFP or MDA-MB-231 cells stably expressing LEPR (1 × 10^6^) were injected in contact with the fat pad or alone as control groups. Tumor growth was directly measured using a caliper. After 8 weeks, tumor metastasis was assessed by bioluminescent imaging on the In Vivo Imaging System (IVIS, Caliper Life Science, Waltham, MA, USA) and then mice were sacrificed. Lungs, livers, and tumors were fixed overnight in 4% paraformaldehyde and embedded in paraffin for H&E staining or immunohistochemistry analysis. Images of these samples were obtained using a BX53 microscope (Olympus, Tokyo, Japan) at 4× and 10× magnification. Metastatic nodules in liver and lung were quantified in five random fields per H&E slide per mice at 4× magnification and are presented as the means ± SEMs (*n* = 6).

To analyze the effect of PAI-1 on tumor metastasis, MDA-MB-231 cells (ShPAI-1 and NC, 1 × 10^6^) were orthotopically injected into the inguinal mammary fat pad of NOD/SCID mice, six mice per group. After 7 weeks, whole-animal bioluminescent imaging was performed using the IVIS system and then the mice were sacrificed, followed by the examination of liver or lung metastases by using H&E staining.

### 2.9. RNA Isolation and qRT-PCR

Total RNA was isolated using the TRIzol Reagent (Vazyme, Nanjing, China) and cDNA was generated from 1 μg total RNA per sample using HiScript qRT SuperMix (Vazyme) and miRNA 1st Strand cDNA Synthesis Kit (Vazyme), respectively for mRNA and mature miRNA. qRT-PCR was performed by using the SYBR Green master mix (Vazyme) and commercially available primers, listed in Table S2.

### 2.10. Proteins Extraction and Western Blot Analysis

Western blotting was performed using whole-cell lysates of breast cancer cells supplemented with phosphatase and protease inhibitors (Beyotime, Shanghai, China). 20μg proteins were separated by 8–12% SDS-PAGE, transferred onto PVDF membranes (Millipore, Burlington, MA, USA), blocked with 5% BSA (BSA dilution in 1× TBST), and incubated with primary antibodies listed in Table S3 for overnight at 4 °C. Signals from HRP-coupled secondary mouse or rabbit antibodies (Abcam, Cambridge, UK) were generated by Chemiluminescent HRP Substrate (Millipore, Burlington, MA, USA).

The TCA precipitation method was performed to analyze secreted proteins in the conditioned medium as previously described [35]. 0.5–1 mL conditioned medium was collected and trichloroacetic acid (Macklin, China) was added to a final concentration of 10%, then vortexed for 1min and incubated on ice for 24 h. The samples were centrifuged at 13000 rpm for 20 min at 4 °C, and the supernatant was discarded and washed twice using 100% ice-cold acetone. Subsequently, dried at 37 °C, followed by dissolved in 50 μL 1× loading buffer and heated for 10 min at 95 °C. 10 μL proteins were separated by 10% SDS-PAGE and followed as described above. 

### 2.11. Immunofluorescence and Immunohistochemistry Assay

For immunofluorescence(IF), cells cultured on glass cover slides were fixed with 4% paraformaldehyde for 30 min, permeabilized in 0.3% Triton X-100 for 15 min, and blocked in 3% BSA/PBS for 1 h. After incubated with the primary antibodies, listed in Table S3, overnight at 4 °C, cells were incubated with the mouse or rabbit secondary antibody, and chromatin was stained with Hoechst for 1 h at room temperature. Confocal laser scanning microscopy images were captured with an FV1000 microscope (Olympus, Tokyo, Japan). For immunohistochemistry (IHC), 5 μm sections of formalin-fixed, paraffin-embedded tissue were used. PAI-1(Abcam, dilution 1:200), E-cadherin (dilution 1:200, Cell Signaling Technologies, Danvers, MA, USA), *n*-cadherin (Cell Signaling Technologies, dilution 1:100), and ORR (dilution 1:200,Abcam,) were used as the primary antibodies, and sections were visualized with DAB detection and hematoxylin and eosin (H&E) staining.

### 2.12. Databases

Metastatic scores of protein factors in cancer were evaluated through Cancer Hallmarks Analytics Tool (CHAT, http://chat.lionproject.net/). The gene expression in breast cancer cell lines was analyzed based on data from the CCLE database (Cancer Cell Line Encyclopedia, https://portals.broadinstitute.org/ccle). The cytokine levels in the human plasm were determined by the analysis of data from the HPA database (The Human Protein Atlas, https://www.proteinatlas.org/). Differential expression of PAI-1 between breast cancer and normal tissues was determined by TCGA (http://cancergenome.nih.gov/) and Ma Breast 4 dataset in Oncomine (https://www.oncomine.org/resource/main.html). Using KM plotter analysis (http://www.kmplot.com/analysis/index.php?p%20=%20background), the prognosis value of PAI-1 and miR-34a was evaluated. In addition, the correlation between genes in cancer was explored by Starbase (http://starbase.sysu.edu.cn/). Putative miRNAs targeting PAI-1 were predicted by TargetScan (http://www.targetscan.org/vert_71/) and miRcode (http://mircode.org/index.php) databases. To identify transcription factors (TFs) upstream of miR-34a, UCSC (http://genome.ucsc.edu/) was used to find promoter sequence of miR-34a, followed by TFs prediction by PROMO (http://alggen.lsi.upc.es/cgi-bin/promo_v3/promo/) and Animal TFBD 3.0 (http://bioinfo.life.hust.edu.cn/AnimalTFDB/#!/predict). Furthermore, potential binding sites of STAT3 on PAI-1 promoter were analyzed by JASPAR (http://jaspar.genereg.net/).

### 2.13. Statistical Analysis

The GraphPad Prism software (University of California San Diego, San Diego, CA, USA) was used to calculate statistical significance. Differences were analyzed by 2-tailed Student’s *t*-test or 2-way ANOVA followed by the Student-Newman-Keuls multiple comparison test in cases in which more than 2 groups were compared in one graph. *p*-value of <0.05 was considered as significant.

## 3. Results

### 3.1. Adipocytes Potentiate Breast Cancer Metastasis by Upregulating PAI-1

**Figure 1:**
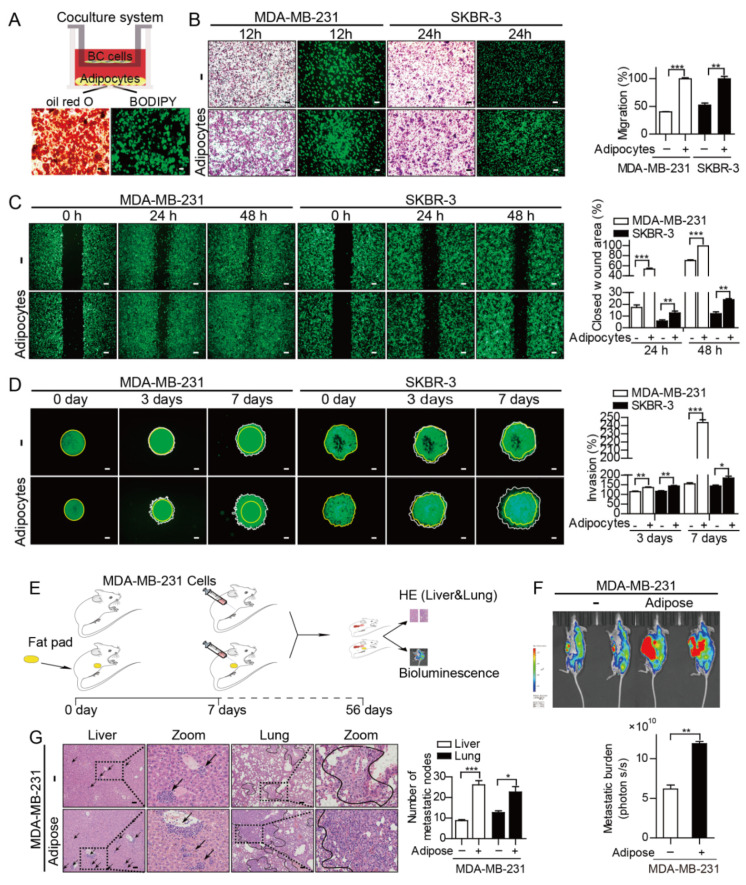
Adipocytes potentiate breast cancer metastasis. (**A**) Coculture system of adipocytes and breast cancer cells (BC cells). (**B**) Transwell migration assays of GFP-labeled cancer cells were performed for 12 h or 24 h after cocultured with adipocytes or monoculture for 72 h. The migratory cells were fixed and stained with a Diff-Quick stain kit. Bars represent the means ± SEMs from three independent experiments. (**C**) The wound healing rate was measured at 24 h and 48 h in MDA-MB-231 and SKBR-3 cells in the presence or absence of adipocytes for 72 h. Bars represent the means ± SEMs from three independent experiments. (**D**) 3D matrix invasion assays were employed to detect the invasion capacity of MDA-MB-231 and SKBR-3 cells on day 3 and day 7 after cocultured with mature adipocytes for 3 days. Yellow and white circles represent the center of the spheroid and the perimeter of cancer cells in a spheroid, respectively. The results are presented as the means ± SEMs from three independent experiments. (**E**) Formation of the breast cancer metastasis model. (**F**) Representative images and quantitative results of bioluminescence in metastatic tumor mice. (**G**) H&E-stained liver and lung sections from the indicated mouse groups. Arrows and circles indicate representative metastases of breast cancer. The number of metastases is shown as the means ± SEMs (*n* = 6). Scale bar = 100 µm. * *p* < 0.05, ** *p* < 0.01, *** *p* < 0.001.

Given that adipocytes represent a dominant part of the breast cancer TME, it is rational to infer that adipocytes contribute to breast cancer progression and metastasis. 3T3-L1 preadipocytes were induced to develop into mature adipocytes, as confirmed by oil red O and BODIPY staining, and then cocultured with breast cancer cells for 72 h (Figure 1A). Firstly, the effect of adipocytes on cancer cell proliferation was evaluated by EdU staining, the results showed that adipocytes slightly contributed to the proliferation of MDA-MB-231 and MCF-7 cells (Figure S1A). Subsequently, the migratory ability of breast cancer cells in the coculture and monoculture groups was estimated by transwell migration and wound healing scratch assays. The cocultured MDA-MB-231 and SKBR-3 exhibited increased migration rates at 12 h and 24 h, respectively (Figure 1B), which were also verified by significant increases in the wound healing ability of MDA-MB-231 and SKBR-3 cells upon adipocyte coculture (Figure 1C). Consistently, adipocytes promoted the invasion of MDA-MB-231 and SKBR-3 cells compared to the invasion of monoculture cancer cells at day 3 and day 7 through 3D invasion assays (Figure 1D). Furthermore, a mouse model of mammary adipose tissue coinjection and GFP-labeled MDA-MB-231 cells was generated to analyze the effects of adipocytes on tumor metastasis in vivo (Figure 1E). After 8 weeks, metastasis in the adipose tissue coinjection groups increased as evidenced by the greater number of metastatic nodules in the livers and lungs than in the control groups (Figure 1F,G). 

**Figure 2:**
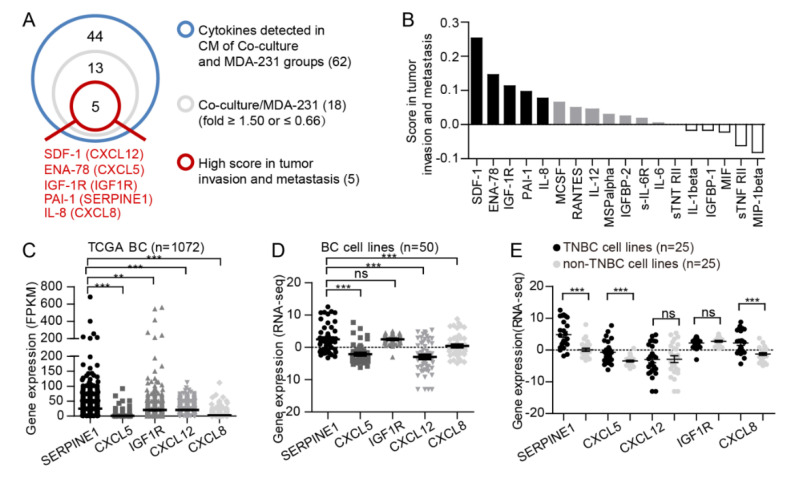

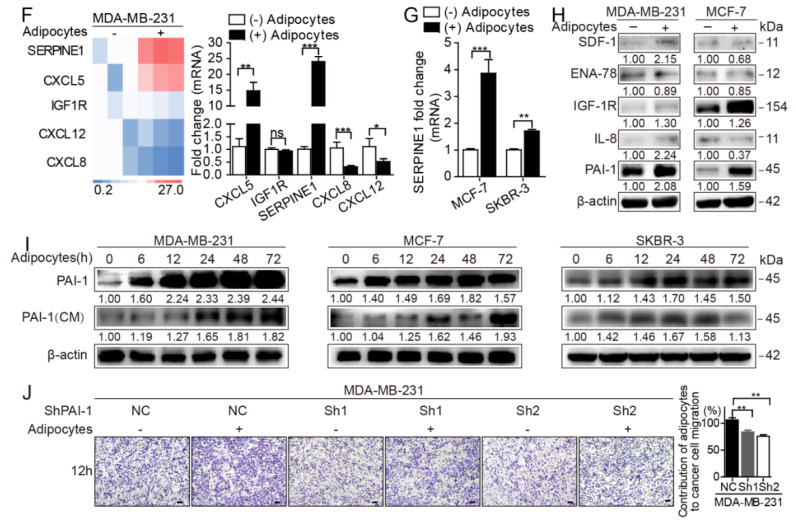
Adipocytes facilitate the migration of breast cancer cells by inducing PAI-1 expression. (**A**,**B**) Analysis of cytokine protein arrays. Among the 62 detected cytokines in the CM of MDA-MB-231 (MDA-231) cells alone or with adipocyte coculture for 72 h, 18 cytokines were initially identified as having a fold change ≥1.50 or ≤0.66 in CM of cocultured cancer cells compared with control groups. Then, the metastatic scores of the above 18 cytokines in cancer were evaluated by the CHAT database (Metric: NPMI), and the top five cytokines were further chosen. (**C**) The expression of the candidate factors in breast cancer (BC) tissues. (**D**) The expression analysis of the candidate factors in breast cancer cell lines. (**E**) The expression of the candidate factors in TNBC and non-TNBC cell lines. (**F**) qRT-PCR assay. MDA-MB-231 cells were cultured ± adipocytes for 72 h, followed by the extraction of RNA and measurement of the expression of the indicated cytokines. Bars represent the means ± SEMs from three independent experiments. (**G**) PAI-1 alteration was explored in MCF-7 and SKBR-3 cells under identical conditions. Bars represent the means ± SEMs from three independent experiments. (**H**) Western blot. The indicated proteins were detected in cancer cells with or without adipocyte coculture for 72 h (*n* = 3 independent experiments). (**I**) Western blot. Different cancer cell lines were cocultured with adipocytes for 0 h to 72 h, and the levels of PAI-1 in cancer cells and CM from the transwell inserts were detected by immunoblotting (*n* = 3 independent experiments). (**J**) MDA-MB-231 cells with stable knockdown of PAI-1 were cultured ± adipocytes for 72 h, and transwell migration assays were employed to estimate the contribution of adipocyte-induced cancer cell migration via PAI-1. Bars represent the means ± SEMs from three independent experiments (* *p* < 0.05, ** *p* < 0.01, *** *p* < 0.001, ns: no significance). Scale bar = 100 µm.

Our previous findings showed that the concentration of some cytokines in the CM of the adipocyte-MDA-MB-231 cell coculture groups was significantly altered compared with that of the MDA-MB-231 monoculture cell groups [15]. Therefore, it was speculated that adipocytes might influence the expression of specific secretory and metastasis-related factors in breast cancer to facilitate tumor metastasis. Based on the cytokine protein arrays, 18 among 62 cytokines were identified to be characterized by a fold change ≥1.5 or ≤0.66 in CM of coculture groups versus monoculture MDA-MB-231 cells (Table S4). Next, the scores of the 18 selected protein factors in tumor metastasis and invasion were determined through the Cancer Hallmarks Analytics Tool (CHAT) [36]. The top five candidate factors, including SDF-1 (CXCL12), ENA-78 (CXCL5), IGF-1R (IGF1R), PAI-1 (SERPINE1), and IL-8 (CXCL8), with higher metastatic scores, were further selected (Figure 2A,B). Among these factors, the highest plasm PAI-1 level was observed in healthy subjects (Figure S1B). Besides, one previous study showed that plasm PAI-1 level was higher in breast cancer patients compared with healthy controls [37]. Interestingly, the differential analyses of these cytokines revealed that PAI-1 was significantly higher in breast cancer tissues compared with normal controls, while lower levels (CXCL5 and CXCL12) or no significant difference (IGF1R and CXCL8) of other cytokines were observed in breast cancer tissues versus normal tissues (Figure S1C). Then the expression of these candidate factors in breast cancer tissues and cell lines were further analyzed based on the data from TCGA and CCLE databases. SERPINE1 was observed with the highest level in tumor tissues among these factors (Figure 2C). Consistently, higher SERPINE1 expression was detected in breast cancer cell lines compared with the other four candidate factors (Figure 2D). Moreover, SERPINE1, CXCL5, and CXCL8 were found dramatically elevated in triple-negative breast cancer (TNBC) cell lines compared with non-triple-negative breast cancer (non-TNBC) cell lines (Figure 2E). Further study showed that SERPINE1 in MDA-MB-231 cells was the most significantly elevated factor at the mRNA level upon adipocyte coculture (Figure 2F), which was also verified in MCF-7 and SKBR-3 cells (Figure 2G). Consistently, upregulation of PAI-1 was detected in MDA-MB-231 and MCF-7 cells upon adipocyte coculture, while the alteration of the other four factors was inconsistent in the two cancer cell lines or inconsistent with the alteration at the mRNA level (Figure 2H). Moreover, the PAI-1 protein extracted from breast cancer cells or CM in transwell inserts showed a time-dependent increase upon adipocyte coculture (Figure 2I). To further assess the potential roles of PAI-1 in breast cancer metastasis induced by adipocytes, PAI-1-knockdown MDA-MB-231 cells (Sh1, Sh2) were generated and cocultured with adipocytes for 72 h followed by migration assays (Figure S1D). 

The results indicated that PAI-1 could contribute approximately 25% to adipocyte-induced breast cancer migration (Figure 2J). More importantly, our previous study demonstrated that depletion of PAI-1 by shRNA attenuated adipose tissue -induced metastasis of breast cancer cells [15].

### 3.2. PAI-1 Induces EMT and the Metastatic Properties of Breast Cancer Cells

**Figure 3:**
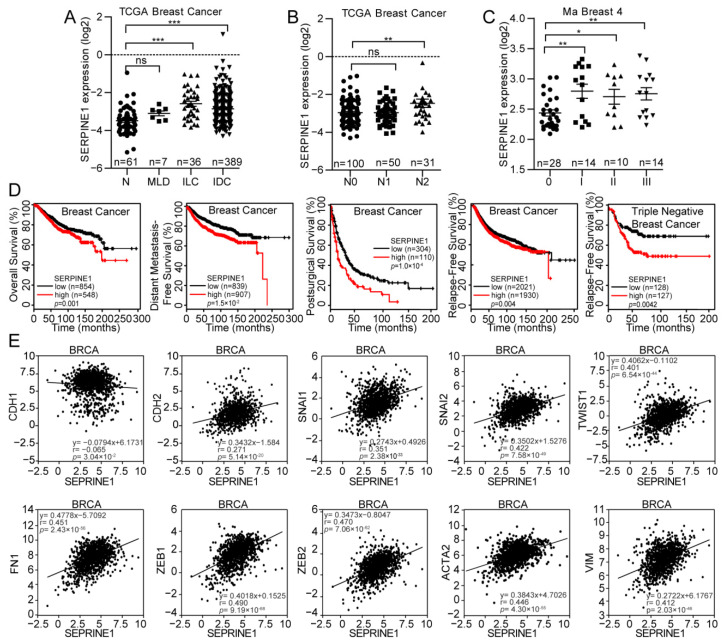
Clinical significance of PAI-1 in patients with breast cancer. (**A**) In accordance with data from the TCGA dataset, PAI-1 in invasive lobular breast carcinoma (ILC) and invasive ductal breast cancer (IDC) was significantly elevated compared with that in normal tissues (N), while no significant difference observed between mix lobular and ductal breast cancer (MLD) and normal tissues. (**B**) PAI-1 is more highly expressed in patients with grade N2 breast cancer than in patients with grade N0 breast cancer. (**C**) PAI-1 in advanced breast cancer was increased compared with that in early breast cancer based on data obtained from Oncomine. (**D**) High PAI-1 expression predicted short distant metastasis-free survival, overall survival (OS), and relapse-free survival (RFS) by the analysis of data from the KM plotter. (**E**) PAI-1 is tightly associated with mesenchymal features in breast cancer, as confirmed by a significantly positive correlation between PAI-1 and mesenchymal markers, including Snail (SNAI1), TCF8 (ZEB1), N-cadherin (CDH2), Twist (TWIST1), Vimentin (VIM), Fibronectin (FN1), Slug (SNAI2) and SIP-1 (ZEB2), while a negative correlation was observed between PAI-1 and E-cadherin (CDH1). * *p* <  0.05, ** *p*  <  0.01, *** *p*  <  0.001, ns: no significance.

The above results support PAI-1 as a key driver in adipocyte-induced metastatic behavior of breast cancer cells. Consequently, the clinical correlation of PAI-1 in breast cancer was further analyzed via clinical databases. Interestingly, significantly higher PAI-1 expression was detected in invasive lobular breast carcinoma (ILC) and invasive ductal breast carcinoma (IDC) than in normal tissues (N), although no significant difference was found between mixed lobular & ductal breast carcinoma (MLD) and normal tissues by analysis of TCGA database (Figure 3A). In addition, PAI-1 expression in N2 stage breast cancer patients increased compared with that in N0 stage breast cancer patients according to the TCGA database (Figure 3B). Similarly, breast cancer patients with high grades appeared to exhibit elevated expression of PAI-1 through the Ma Breast 4 database (Figure 3C). More importantly, Kaplan-Meier analysis indicated that PAI-1 might serve as an indicator of poor prognosis in breast cancer, as evidenced by elevated PAI-1 expression being associated with unfavorable overall survival (OS), distant metastasis-free survival (DMFS), and postsurgical survival (PS). Interestingly, PAI-1 was also a significant prognostic factor in relapse-free survival (RFS) for patients with triple-negative breast cancer (Figure 3D). In terms of previous investigations, PAI-1 might serve as an independent predictor for breast cancer, although a positive correlation has also been found between PAI-1 and traditional prognostic factors, such as tumor size, grade, stage, and pathohistological type [38]. 

**Figure 4:**
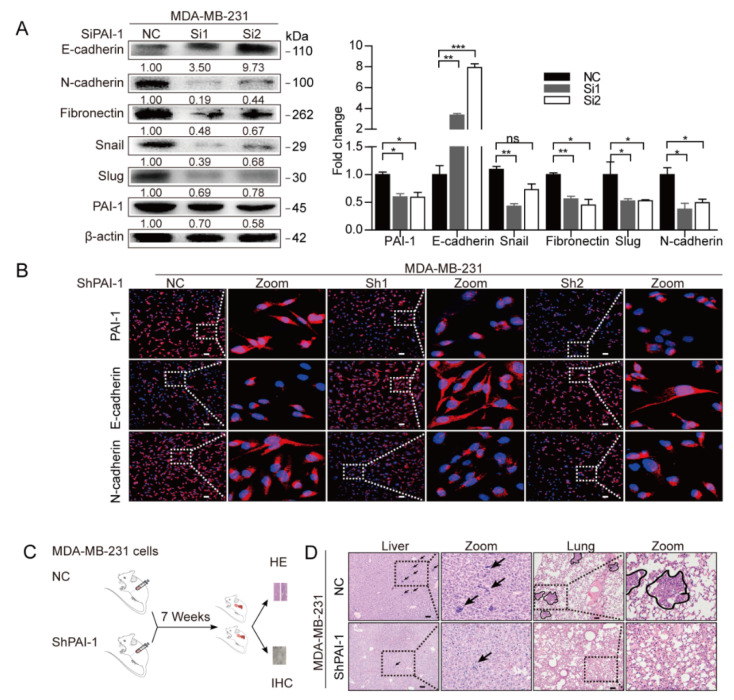

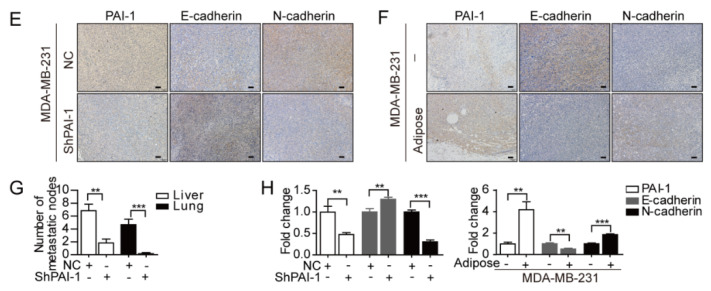
Depletion of PAI-1 blocks EMT and metastasis of breast cancer cells. (**A**) Western blot analysis after depletion of PAI-1 in MDA-MB-231 cells with SERPINE1-specific siRNA to detect alterations in EMT-related markers. Error bars represent the means ± SEMs (*n* = 3 independent experiments). (**B**) Representative images of immunofluorescence for the indicated proteins in PAI-1-stable knockdown MDA-MB-231 cells and control groups. (**C**) Orthotopic xenograft tumor formation in NOD/SCID mice with PAI-1 stable silencing MDA-MB-231 cells (ShPAI-1) or negative control cancer cells (NC). After 7 weeks, mice were sacrificed to collect livers, lungs, and tumors for H&E staining (**D**) and IHC. Arrows and circles indicate representative metastases. (**E**) The IHC for PAI-1, E-cadherin, and N-cadherin was performed on subcutaneous tumors from shPAI-1 or NC cancer cell groups. (**F**) Representative IHC analyses of PAI-1, E-cadherin, and N-cadherin in tumors from mice coinjection with adipose tissue and cancer cells or mice injected with cancer cells alone. (**G**) Quantitative results of the metastases (*n* = 6). (**H**) Quantitative results of the IHC (*n* = 6). Scale bar: 100 µm. * *p* < 0.05, ** *p* < 0.01, *** *p* < 0.001.

Recently, the critical contribution of EMT to cancer metastasis has been highlighted in advanced research [39]. However, the role of PAI-1 in the EMT process of breast cancer remains unclear, therefore, the TCGA database was used to assess the correlation between PAI-1 and EMT markers. Notably, a positive correlation was identified between PAI-1 and multiple mesenchymal cell markers, while PAI-1 was inversely associated with epithelial cell markers in breast cancer (Figure 3E). To further verify the effect of PAI-1 on the EMT process in breast cancer cells, PAI-1 was depleted with siRNA or stably silenced in MDA-MB-231 cells and the knockdown efficiency was determined by qRT-PCR (Figure S1), followed by western blot or immunofluorescence analysis to determine the alteration in EMT-related markers. As expected, depletion of PAI-1 resulted in an epithelial-like phenotype in MDA-MB-231 cells, as manifested by a marked increase in E-cadherin and a decrease in mesenchymal cell markers such as fibronectin, Snail, and Slug (Figure 4A,B). Subsequently, in vivo profiling revealed that stable knockdown of PAI-1 attenuated liver and lung metastasis (Figure 4C,D,G), consistent with the EMT markers in PAI-1-deficient groups being altered compared with those in control groups (Figure 4E,H). Since adipocytes have been validated to stimulate breast cancer metastasis in vivo (Figure 1E–G) and adipocytes could increase PAI-1 expression in breast cancer cells (Figure 2C–E), the expression of the PAI-1 and EMT markers was detected in these samples. Higher expression of PAI-1 and N-cadherin, while lower expression of E-cadherin, was observed in adipose tissue-cancer cell coinjection groups compared to in control groups (Figure 4F,H), suggesting that adipocytes could induce PAI-1-mediated EMT to promote breast cancer metastasis.

### 3.3. Adipocyte-Derived Leptin Facilitates PAI-1-Mediated Breast Cancer Metastasis

**Figure 5:**
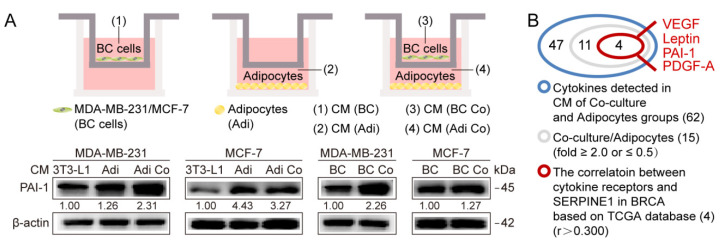

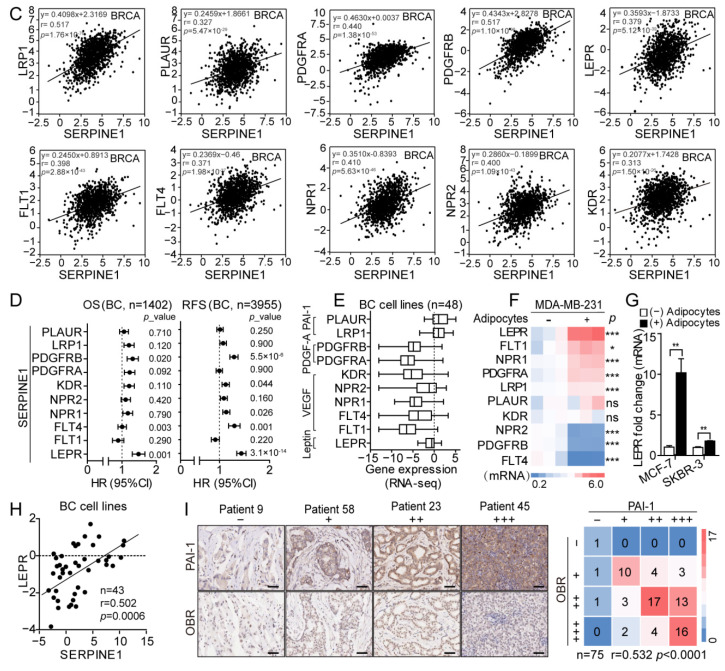
Activation of OBR is associated with adipocyte-induced PAI-1 expression. (**A**) The indicated cancer cells were treated with CM from cancer cells or adipocytes under monoculture or coculture conditions for 72 h, and western blot analysis was performed for PAI-1 expression (*n* = 3 independent experiments). (**B**–**C**) Analysis of cytokine protein arrays. Among all the detected factors, 15 adipokines were initially identified as fold change ≥2.0 or ≤0.5 in CM of cocultured groups compared with that of adipocyte groups. Based on the correlation (r > 0.3) between PAI-1 and receptors of the 15 adipokines in breast cancer via TCGA dataset, 10 receptors and their counterpart adipokines including VEGF, leptin, PAI-1, and PDGF-A were further chosen. (**D**) The hazard ratio (HR) for OS and RFS of breast cancer by the expression of PAI-1 together with adipokine receptors. (**E**) The basic expression of adipokine receptors in breast cancer cell lines. (**F**) MDA-MB-231 cells were cultured ± adipocytes for 72 h and expression of the 10 receptors was analyzed by qRT-PCR. (**G**) LEPR expression was detected in SKBR-3 and MCF-7 cells under identical culture conditions. Error bars represent the means ± SEMs from three independent experiments. (**H**) The correlation of LEPR and SERPINE1 in breast cancer cell lines. (**I**) The expression of OBR and PAI-1 was detected in 75 breast cancer samples by IHC. The staining was divided into 4 grades (0, 1+, 2+, and 3+) based on the staining intensity. Scale bar: 100 µm. * *p*< 0.05, ** *p* < 0.01, *** *p* < 0.001, ns: no significance.

Given the crucial roles of adipokine-mediated communication between adipocytes and cancer cells, it was hypothesized that adipocyte-derived adipokines might affect PAI-1 expression in breast cancer cells upon binding to the associated receptors. CM was collected from the coculture system of adipocytes and breast cancer cells, monoculture adipocytes and breast cancer cells, as well as 3T3-L1 preadipocytes, followed by supporting the growth of breast cancer cells for 72 h, then, samples were collected for western blot analysis (Figure 5A). Notably, compared with CM from 3T3-L1 cells, CM from adipocytes alone or cocultured adipocytes facilitated the expression of PAI-1 in MDA-MB-231 and MCF-7 cells (Figure 5A, Figure S1E). Additionally, breast cancer cells showed higher PAI-1 expression with the stimulation of CM from coculture cells than from monoculture tumor cells (Figure 5A, Figure S1E). 

To identify the adipokines involved in PAI-1 regulation, cytokine protein arrays were performed as previously described [15]. Fifteen adipokines were initially found to be characterized by a significant fold change ≥2.0 or ≤0.5 in the CM of cocultured groups compared with the CM of adipocytes alone (Table S5). Given adipokines function upon binding to corresponding receptors, a positive correlation can exist between PAI-1 and the adipokine receptor in cancer cells. Therefore, all counterpart receptors of the identified adipokines were determined, followed by correlation analysis between the receptors and PAI-1 in breast cancer by using the TCGA dataset (Table S6). In terms of the correlation coefficient r > 0.3, the top 10 adipokine receptors and their counterpart adipokines including VEGF, leptin, PAI-1, and PDGF-A were identified (Figure 5B,C). Next, the prognostic value of PAI-1 together with the adipokine receptor was analyzed via Kaplan-Meier analysis. Interestingly, high expression of PAI-1 and LEPR was accompanied by the highest hazard ratio for OS and RFS of breast cancer among all the combinations of PAI-1 and adipokine receptor (Figure 5D). In addition, the expression of these receptors was analyzed in breast cancer cell lines, a higher level of LEPR was observed in breast cancer cell lines compared with other adipokine receptors, except for the PAI-1 receptors (Figure 5E). Further studies revealed that the leptin receptor (termed LEPR/OBR) in MDA-MB-231 cells was the most strongly upregulated receptor at the mRNA level in the presence of adipocytes for 72 h. Likewise, a LEPR increase was observed in cocultured MCF-7 and SKBR-3 cells (Figure 5F,G). Moreover, adipocytes could induce OBR expression in breast cancer cells at the protein level [16]. Further correlation analysis suggested that PAI-1 was positively associated with LEPR in different breast cancer cell lines (Figure 5H). Consistently, the positive correlation between PAI-1 and LEPR was validated in clinical breast cancer specimens (Figure 5I). Leptin is generally accepted to function via OBR. In addition, our previous study indicated that leptin was expressed at much higher levels in adipocytes than 3T3-L1 preadipocytes, and the crosstalk between adipocytes and breast cancer cells constituted a feedback system to sustain high levels of leptin in the coculture system [16]. Besides, the leptin (LEP) level was significantly positively correlated with LEPR and PAI-1 levels in breast cancer tissues via the TCGA dataset (Figure S1F). Based on the above analysis, we further focused on the potential regulation of PAI-1 by leptin/OBR.

**Figure 6:**
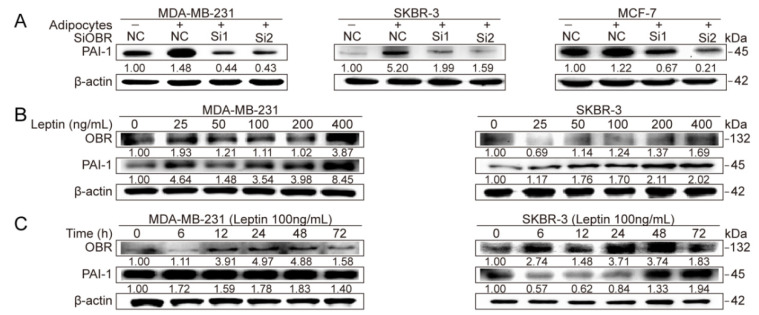

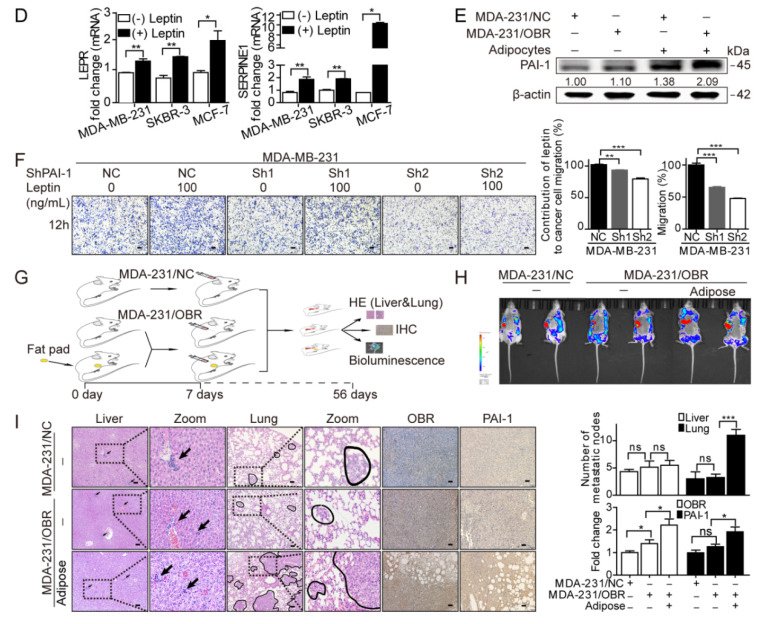
Adipocyte-derived leptin induces PAI-1-mediated breast cancer metastasis by activating OBR. (**A**) Breast cancer cells transfected with OBR-specific siRNA or NC siRNA were cultured ± adipocytes for 72 h, and western blotting was performed for the indicated proteins (*n* =3 independent experiments). (**B**,**C**) Western blot. The indicated proteins were detected after treatment with leptin at different doses and time points (*n* = 3 independent experiments). (**D**) Expression of LEPR and SERPINE1 was measured by qRT-PCR (*n* = 3 independent experiments). (**E**) PAI-1 expression was determined after negative control MDA-MB-231 cells (MDA-231/NC) or MDA-MB-231/OBR cells (MDA-231/OBR) were cultured ± adipocytes for 72 h via western blot. (**F**) MDA-MB-231 cells with stable knockdown of PAI-1 were exposed to leptin and analyzed with migration assays. Quantitative results of the migration assays are from 3 independent experiments. (**G**) Formation of the metastasis model. (**H**) Representative images of bioluminescence, the quantification results were shown in Figure S1H. (**I**) H&E staining and IHC analysis. Arrows and circles indicate representative metastatic nodes. Scale bar: 100 µm. * *p* < 0.05, ** *p*  < 0.01, *** *p* < 0.001, ns: no significance.

First, OBR-specific siRNA was utilized to impair OBR expression in breast cancer cell lines followed by cocultured with adipocytes. The silencing efficiency of OBR-specific siRNA was determined at the mRNA level (Figure S2A,B). Strikingly, OBR depletion attenuated adipocyte-induced PAI-1 expression in MDA-MB-231, SKBR-3, and MCF-7 cells (Figure 6A, Figure S2C). Also, MDA-MB-231 and SKBR-3 cells were exposed to leptin, at a concentration ranging from 25 ng/mL to 400 ng/mL for 24 h, or 100 ng/mL leptin stimulation for 0 h to 72 h, respectively. PAI-1 was increased by leptin in a dose-dependent manner, while no significant time-dependent increase was observed (Figure 6B,C, Figure S2D,E). Moreover, with 100 ng/mL leptin treatment for 72 h, OBR and PAI-1 were also elevated at the mRNA level in MDA-MB-231, SKBR-3, and MCF-7 cells (Figure 6D). Consistently, ectopic expression of OBR slightly increased PAI-1 expression in cancer cells, and OBR overexpression markedly strengthened adipocyte-induced PAI-1 expression (Figure 6E, Figure S2F,G). Subsequently, PAI-1- knockdown cancer cells were exposed to leptin (100 ng/mL, 72 h) and measured with migration assays for 12 h. These results indicate that leptin promoted the migration of MDA-MB-231 cells, while knockdown of PAI-1 abrogated leptin-induced migration of MDA-MB-231 cells (Figure 6F). Further in vivo investigation indicated that a higher level of PAI-1 was detected in OBR-overexpression tumors (MDA-231/OBR) compared with control groups (MDA-231/NC), with no significance. 

Besides, adipose tissue coinjection significantly induced PAI-1 expression in MDA-231/OBR tissues (Figure 6I). Meanwhile, coinjection of adipose tissues potentiated lung metastasis of MDA-231/OBR cells (Figure 6G–I, Figure S2H). Collectively, these findings imply that adipocyte-derived leptin activated OBR to modulate PAI-1-induced breast cancer metastasis.

### 3.4. Repression of MiR-34a Contributes to Leptin/OBR-Induced PAI-1 Expression 

Next, we sought to explore the molecular mechanisms whereby PAI-1 was modulated by adipocyte-derived leptin. To date, the upstream regulators of PAI-1 can be mainly classified into cytokines, growth factors, hormones, microRNAs (miRNAs), and specific environmental stress [40]. miRNAs usually downregulate targets at the protein level by repressing translation by binding to the 3′-UTR of the target mRNA [41], or by inducing mRNA degradation [42]. Leptin regulated PAI-1 expression at the protein and mRNA levels. Therefore, we hypothesized that certain PAI-1-targeting miRNAs might exist downstream of leptin. 

**Figure 7:**
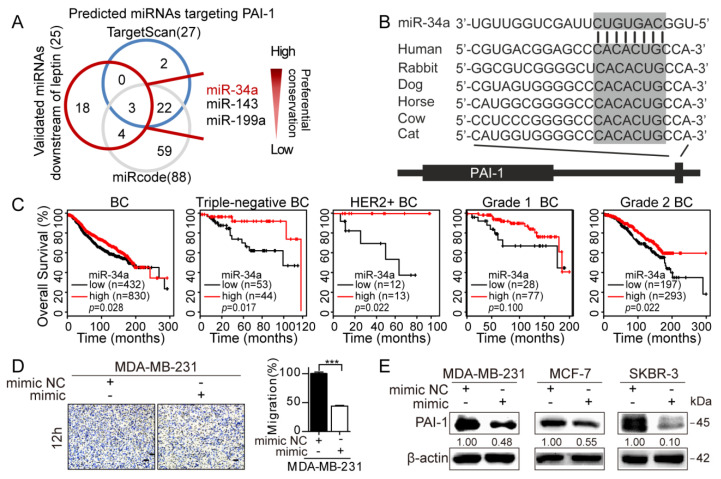

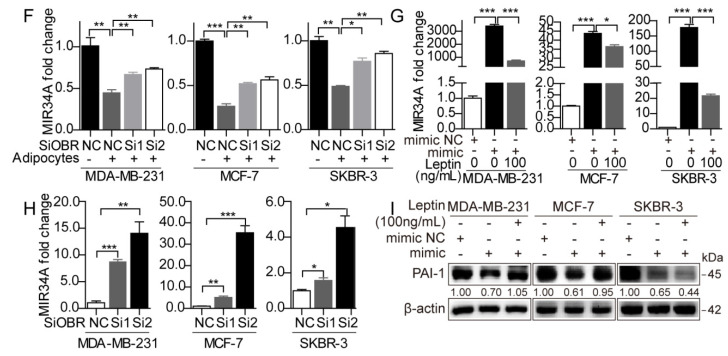
Leptin/OBR induces PAI-1 expression by repressing miR-34a. (**A**) Prediction of miRNAs targeting PAI-1. A total of 25 potential PAI-1-targeting miRNAs were simultaneously predicted by TargetScan and miRcode databases. (**B**) Conservative binding site for miR-34a at the 3′UTR of PAI-1. (**C**) Prognostic value of miR-34a in patients with different types or grades of breast cancer by KM plotter analysis. (**D**) Transwell migration assays of MDA-MB-231 cells after transfection with the miR-34a mimic (100 nM, 48 h). Scale bar: 100 µm. (**E**) Breast cancer cells transfected with the miR-34a mimic (100 nM, 48 h) were collected for western blot analysis. (**F**) qRT-PCR. OBR was depleted in breast cancer cells with siRNA followed by adipocyte coculture for 72 h, and then miR-34a expression was detected. (**G**) Expression of miR-34a was measured in breast cancer cells by qRT-PCR after transfection with the miR-34a mimic (100 nM, 48 h) and treatment with leptin (100 ng/mL, 24 h). (**H**) Breast cancer cells transfected with OBR-specific siRNA were collected for qRT-PCR to detect miR-34a expression. (**I**) After transfection with the miR-34a mimic (100 nM, 48 h), breast cancer cells were further treated with leptin (100 ng/mL, 24 h), and then the PAI-1 levels were examined by western blot. The results are presented as the mean ± SEMs from three independent experiments, * *p* < 0.05, ** *p* < 0.01, *** *p* < 0.001.

The TargetScan and miRcode databases were utilized to predict potential miRNAs that targeted PAI-1 (Table S7). Three candidate miRNAs, including miR-34a, miR-143, and miR-199a, were initially predicted by both databases and previously validated downstream of leptin [43,44,45]. Then miR-34a was further examined (Figure 7A). First, TargetScan prediction revealed that miR-34a has the most preferential binding sites at the PAI-1 3′-UTR among the three miRNAs (Figure 7A,B). In addition, a negative correlation between PAI-1 and miR-34a was observed in different breast cancer cell lines (Figure S3A). Further survival analysis indicated that low miR-34a expression predicted poor outcome in patients with breast cancer, and consistent results were observed in different subtypes and high-grade breast cancers (Figure 7C). These results imply that miR-34a functions as a tumor suppressor in breast cancer. miR-34a was overexpressed with miR-34a mimic (100 nM, 48 h) and evaluated by qRT-PCR (Figure S3B). As expected, the migration ability of MDA-MB-231 cells was significantly attenuated after transfection with the miR-34a mimic (Figure 7D). In addition, the miR-34a mimic strongly reduced PAI-1 expression at the protein level in different breast cancer cell lines, whereas no significant change was detected at the mRNA level (Figure 7E, Figure S3C,D). Similarly, PAI-1 was verified to be a target of miR-34a in A549 and H520 cells through western blot and luciferase reporter assays, while PAI-1 showed no significant alteration at the mRNA level when miR-34a was overexpressed [33].

Subsequently, the effect of adipocyte-derived leptin on miR-34a expression in cancer cells was evaluated. Notably, the coculture breast cancer cells exhibited markedly lower expression of miR-34a than the monoculture cancer cells, while depletion of OBR partially reversed the inhibitory effect of adipocytes on miR-34a (Figure 7F). In addition, miR-34a mimic-induced elevation of miR-34a in cancer cells was significantly abrogated after treatment with leptin (Figure 7G). In contrast, silencing of OBR led to an increase in the miR-34a level in breast cancer cells (Figure 7H). Furthermore, the inhibitory effect of the miR-34a mimic on PAI-1 was reversed in different cancer cells following leptin treatment (Figure 7I, Figure S3E). Together, these data suggest that miR-34a repression was crucial for leptin/OBR induced PAI-1 expression in the interaction between adipocytes and breast cancer cells.

### 3.5. STAT3 Mediates the Leptin/OBR Induced Repression of miR-34a and Activation of PAI-1

Leptin was confirmed to enhance PAI-1 expression in breast cancer cells at both the protein and mRNA levels (Figure 6B–D). However, miR-34a mainly mediated the effect of leptin on PAI-1 at the protein level rather than at the mRNA level (Figure 7E, Figure S3C,D). These results led us to focus on transcription factors (TFs), which act at the transcriptional level. Interestingly, TFs have also been considered regulators of miR-34a in cancer [46]. Therefore, we speculated that certain TFs might exist downstream of leptin/OBR and upstream of PAI-1, which could not only directly affect the transcription of PAI-1 but also indirectly regulate PAI-1 expression via miR-34a. 

**Figure 8:**
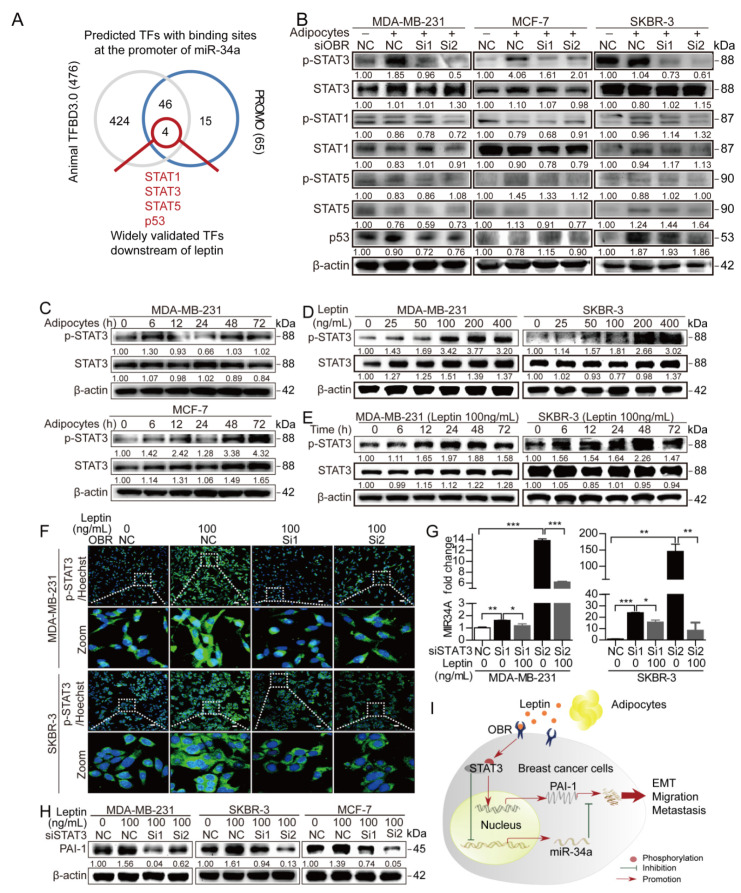
STAT3 is involved in leptin/OBR-induced miR-34a suppression and PAI-1 activation. (**A**) Prediction of TFs with binding sites at the miR-34a promoter. (**B**) Cancer cells were transfected with OBR-specific siRNA or NC siRNA followed by culture ± adipocytes for 72 h, and then western blotting was performed. (**C**) Cancer cells were cocultured with adipocytes for the indicated time points followed by western blotting. (**D**,**E**) After stimulation with leptin (0–400 ng/mL for 24 h or 100 ng/mL for 0–72 h), cancer cells were collected for western blotting. (**F**) Immunofluorescence for p-STAT3 in cancer cells after transfection with OBR-specific siRNA or NC siRNA followed by leptin treatment (100 ng/mL, 72 h). Scale bar = 100 µm. (**G**) After transfection with STAT3-specific siRNA, cancer cells were treated with leptin (100 ng/mL, 24 h) and then collected for qRT-PCR. Bars indicate means ± SEMs from three independent experiments, * *p* <  0.05, ** *p*  <  0.01, *** *p*  <  0.001. (**H**) STAT3-specific siRNA or NC siRNA was transfected into cancer cells followed by exposure to leptin (100 ng/mL, 24 h), and then western blotting was performed. (**I**) Schematic diagram of the proposed mechanisms whereby adipocytes induced metastasis of breast cancer cells.

First, UCSC (http://genome.ucsc.edu/) was utilized to determine the promoter sequence of miR-34a and then putative TFs binding to the miR-34a promoter were predicted by the PROMO and Animal TFBD 3.0 databases (Table S8). Among the fifty TFs predicted by both databases, STAT1, STAT3, STAT5 [47], and p53 [48] were initially selected as previously validated downstream factors of leptin (Figure 8A). To discover which TF mainly exerted the downstream effects of leptin/OBR in the coculture system, OBR was silenced with OBR-specific siRNA followed by adipocyte coculture. Interestingly, p-STAT3 showed significant and consistent alterations in the three breast cancer cell lines under the above conditions, while the other three TFs remained unchanged or inconsistent alterations (Figure 8B, Figure S3F). In addition, ectopic OBR expression slightly stimulated the activation of p-STAT3 in MDA-MB-231 cells, while this effect of OBR was strengthened after cocultured with adipocytes for 72 h (Figure S4A). Furthermore, p-STAT3 was elevated in MDA-MB-231 and MCF-7 cells in a time-dependent manner with adipocyte coculture (Figure 8C, Figure S4B). Additionally, leptin induced the activation of p-STAT3 in MDA-MB-231, and SKBR-3 cells in a dose- and time-dependent manner (Figure 8D,E, Figure S4B–D). Likewise, p-STAT3 accumulated in the nuclei of MDA-MB-231, SKBR-3 and MCF-7 cells following leptin treatment, while this accumulation was reversed by depletion of OBR (Figure 8F, Figure S4E). These results imply that STAT3 activation downstream of leptin/OBR is crucial in the interaction between adipocytes and cancer cells.

To assess STAT3 as a mediator between leptin and miR-34a, STAT3 was depleted with siRNAs and verified at the protein level (Figure S4F) followed by leptin treatment. Strikingly, STAT3 depletion-induced miR-34a upregulation was abrogated by further leptin stimulation in cancer cells (Figure 8G), implying that miR-34a is a signaling factor mediating the indirect effect of STAT3 on PAI-1 downstream of leptin/OBR. Next, the direct effect of STAT3 on PAI-1 expression was explored. Notably, the leptin-induced increase in PAI-1 in breast cancer cells was significantly blocked by silencing STAT3 (Figure 8H). Interestingly, conserved STAT3 binding sites were observed in the JASPAR database (Figure S4G). Indeed, several putative binding sites of STAT3 in the PAI-1 promoter were predicted by the JASPAR database (Table S9), and these predictions require further investigation. Taken together, leptin mediated crosstalk between adipocytes and breast cancer cells, which further activated STAT3 not only directly to facilitate PAI-1 expression but also to indirectly affect PAI-1 by repressing miR-34a, thus promoting PAI-1-related EMT to strengthen breast cancer metastasis (Figure 8I, Figure S4H).

## 4. Discussion

PAI-1 is critical for breast cancer development, as evidenced by increasing fibrin deposition, promoting cytoskeletal reorganization, and affecting the EMT process [49,50,51]. Recently, our group found that adipocyte coculture increased the expression and secretion of PAI-1 in breast cancer cells, and the secreted PAI-1 enriched adipocyte-derived linear collagen, which served as a “highway” for cancer cell migration [15]. Nonetheless, the roles of PAI-1 behind adipocyte-driven tumor metastasis in breast cancer cells remain obscure. Mechanistically, growth factors, miRNAs, and cytokines such as TGF-β, EGF, oncostatin M, and miR-30c, have been demonstrated to regulate PAI-1 expression [49,52,53,54]. Given the endocrine function of adipocytes, we speculated that adipocyte-derived cytokines might also modulate PAI-1 expression. In this study, we validated PAI-1 as a novel driver accounting for adipocyte-induced metastatic behavior of breast cancer cells. Mechanistic studies elucidated that adipocyte-derived leptin upregulated PAI-1 expression in a STAT3/miR-34a-dependent manner. Our findings provide new evidence that breast cancer may be treated by targeting PAI-1 and interfering with its upstream regulators in cancer cells and adipocytes.

In this study, we screened for secreted proteins in the co-cultured cancer cells and confirmed leptin as a positive regulator of PAI-1. Other secreted-proteins, such as MPS-1, ICAM-1, and PLOD2, have also been validated as critical downstream effectors of leptin in cancers [16,55,56]. MPS-1 was demonstrated to promote the leptin-induced growth of colorectal cancer via activating the JNK/c-Jun pathway [55]. Another study revealed that leptin provoked the JAK1/2, STAT3, FAK, ERK, and GSK3αβ signaling cascade to stimulate the production of soluble ICAM-1 in cancer cells, which induced osteoclast formation and enhanced tumor-induced osteolysis in vivo [56]. On the other hand, leptin-induced non-secreted proteins, such as OBR, PKM2, ACAT2, and SREBP-1, are also important for breast cancer evolution [57,58,59,60]. Leptin was found to stimulate OBR expression via STAT3/G9a/miR-200c signaling cascade, and OBR promoted the formation of breast cancer stem-like cells (CSC) [57]. PKM2 was another EMT-related protein downstream of leptin in breast cancer [58]. Also, leptin potentiated the proliferation, migration, and invasion of breast cancer cells via upregulating ACAT2 through the PI3K/AKT/SREBP2 signaling pathway [59]. Recently, leptin was observed to stimulate SREBP-1 expression to induce metabolic reprogramming of cancer cells, which is crucial for the growth of breast cancer cells [60]. 

ASCs-derived leptin has been reported to enhance PAI-1 expression in MCF-7 cells [14]. Similarly, we found that adipocyte-derived leptin increased PAI-1 expression in not only MCF-7 cells but also MDA-MB-231 and SKBR-3 cells via upregulating OBR (Figure 6A–E). Consistently, leptin was reported to promote OBR expression by suppressing miR-200c in MCF-7 cells [57]. Although leptin stimulates PAI-1 expression by activating the ERK1/2 pathway in human vascular endothelial cells [61], the detailed molecular mechanisms behind this regulation remain unclear in cancer cells. PI3K/AKT, JAK2/STAT3, and ERK1/2 signaling pathways are well characterized downstream signaling pathways of leptin/OBR [62,63,64]. Our previous study demonstrated that leptin promoted PLOD2-mediated breast cancer metastasis via the activation of PI3K/AKT and JAK/STAT3 signaling pathways, while ruxolitinib (a selective inhibitor of JAK1 and JAK2) and LY294002 (an inhibitor of PI3Ks) reversed leptin-induced activation of these signals [16]. In this study, our data showed that leptin stimulated the phosphorylation of STAT3 and the translocation of p-STAT3 to the nucleus of cancer cells (Figure 8D–F). In addition, depletion of OBR inhibited the leptin-induced accumulation of p-STAT3 in the nucleus of cancer cells (Figure 8F and Figure S4E). Thus, OBR is of great importance to mediate the function of leptin.

It is meaningful to compare the functional impact of the intrinsic PAI-1 and the leptin-induced PAI-1 for cancer cells. The intrinsic PAI-1 is critical for EMT-related breast cancer metastasis (Figure 4). ASCs-derived leptin promoted tumor growth and metastasis of estrogen receptor-positive breast cancer by upregulating PAI-1 and MMP-2 [14]. Consistently, our data revealed that leptin reversed PAI-1 depletion-induced attenuation of cancer cell migration (Figure 6F). Leptin-induced PAI-1 provides evidence for the critical roles of tumor microenvironment in breast cancer malignant evolution by affecting PAI-1 expression. However, to better clarify this question, the downstream mechanisms of PAI-1 need to be well characterized in cancer cells. PAI-1 can exist as tPA-PAI-1, uPA-PAI-1, vitronectin-PAI-1, and LPR1-PAI-1 complexes, which are closely related to the function of PAI-1 in cancers [65]. In addition, H. Mio et al. observed that PAI-1 facilitated invasion and lung metastasis of osteosarcoma cells via promoting MMP-13 expression and secretion [66]. Another study showed that over-expression of PAI-1 accelerated head and neck cancer cell migration by the activation of the PI3K/AKT pathway [67]. Nonetheless, the downstream mechanisms of PAI-1 remain largely unclear. Altogether, this question deserves more attention and effort.

PAI-1, as a target of miR-34a, has been verified in non-small-cell lung cancer (NSCLC) cells by luciferase reporter assays, while overexpression of miR-34a reduced PAI-1 expression at the protein but not mRNA level in A549 and H520 cells [39]. Likewise, we found that the miR-34a mimic significantly decreased PAI-1 expression in MDA-MB-231, MCF-7, and SKBR-3 breast cancer cells at the protein level rather than at the mRNA level (Figure 7E, Figure S3C,D). However, miR-34a mimic markedly reduced PAI-1 expression in MNNG/HOS and MG-63 osteosarcoma cells at the mRNA level [68]. These results suggest that the regulation of PAI-1 by miR-34a might not be identical in cancer cells with different cellular contexts. Consistent with the results of a previous study [36], inhibition of miR-34a by external leptin was confirmed in different breast cancer cells (Figure 7G). Furthermore, our findings revealed that adipocyte-derived leptin inhibited miR-34a expression in breast cancer cells by interacting with OBR (Figure 7F–H). Accordingly, miR-34a repression was critical for leptin/OBR-induced upregulation of PAI-1 in the adipocyte-cancer cell interaction. More importantly, miR-34a-based cancer therapy is not an unreachable aim, since various nanoscale vectors have widely focused on ensuring the efficiency of miR-34 mimics [27].

Consistent with other findings that miR-34a significantly increased with the treatment of stattic (a small-molecule inhibitor of STAT3) [69], we found that depletion of STAT3 markedly attenuated the leptin-induced suppression of miR-34a in breast cancer cells (Figure 8G). Moreover, IL-6-dependent activation of STAT3 was found to directly repress the miR-34a gene in colorectal cancer cells [70]. Intriguingly, our recent findings revealed that adipocyte-derived IL-6 activated the JAK/STAT3 pathway to increase PLOD2 by interacting with GP130 [16]. Thus, IL-6/GP130 might also contribute to STAT3-mediated PAI-1 expression. Nonetheless, leptin/OBR-induced upregulation of PAI-1 is much close to clinical situations than IL-6/GP130 in breast cancer, as the correlation between PAI-1 and OBR (r = 0.379) is much stronger than that between PAI-1 and GP130 (r = 0.064) and between PAI-1 and IL6R (r = 0.162) based on TCGA dataset (Table S6). In addition, we observed that silencing STAT3 blocked the leptin-induced increase in PAI-1 in breast cancer cells (Figure 8H). Several conserved STAT3-binding sites at the promoter of PAI-1 have been predicted by JASPAR (Table S9), and further study is essential to confirm the detailed binding sites involved in STAT3-mediated PAI-1 regulation.

## 5. Conclusions

In the present study, PAI-1 was confirmed as a new driver in adipocyte-driven metastatic behavior of breast cancer cells. Indeed, PAI-1 can serve as a marker to evaluate the metastatic risk of breast cancer, as the positive correlation of PAI-1 and mesenchymal features is well characterized in breast cancer patients. To summarize the results from a clinical perspective, we described that leptin/OBR is tightly related to PAI-1 expression in breast cancer, which is novel evidence reflecting cytokine-mediated communication between adipocytes and breast cancer cells. The detailed mechanisms underlying such regulation remain largely unknown in cancer cells. Herein, our findings reveal that adipocyte-derived leptin interacts with OBR to activate STAT3, thereby directly increasing PAI-1 expression or indirectly regulating PAI-1 by repressing miR-34a. Collectively, our study provides new evidence for combining anticancer therapies to target both cancer cells and adipocytes.

## Supplementary Materials

The following are available online at https://www.mdpi.com/2072-6694/12/12/3864/s1, File S1: Plasmid information of LEPR overexpression, Table S1: The oligonucleotides used in this work, Table S2: Primer sequence for qRT-PCR, Table S3: Antibodies for WB, IF and IHC, Table S4: Relative alteration of cytokines in CM of Co-culture/MDA-MB-231. Table S5: Relative alteration of cytokines in CM of Co-culture/Adipocytes. Table S6: Correlation of PAI-1 and adipokine receptors in breast cancer by the analysis of TCGA dataset. Table S7: Predicted miRNAs targeting PAI-1. Table S8: Predicted transcription factors upstream of miR-34a. Table S9: Potential binding sites of STAT3 at PAI-1 promoter. FigureS1: Quantitative results and loss and gain efficiency of OBR, Figure S2: miR-34a and STAT3 were involved in the regulation of leptin/OBR on PAI-1, Figure S3: Expression of PAI-1 in different breast cancer cell lines, Figure S4: Quantitative results and silence efficiency of STAT3, Figure S5: Expression of PAI-1 in different breast cancer cell lines.

## Abbreviations

TME	Tumor microenvironment
CAFs	Cancer-associated fibroblasts
TAMs	Tumor-associated macrophage
ASCs	Adipose stem cells
CAAs	Cancer-associated adipocytes
ECM	Extracellular matrix
CHAT	Cancer Hallmarks Analytics Tool
CM	Conditioned medium
ILC	Invasive lobular breast carcinom
IDC	Invasive ductal breast carcinoma
MLD	Mixed lobular & ductal breast carcinoma
PAI-1/SERPINE1	Plasminogen activator inhibitor 1
OBR/LEPR	Leptin receptor
IF	Immunofluorescence
IHC	Immunohistochemistry
WB	Western blot
OS	Overall survival
DMFS	Distance metastasis free survival
PS	Postsurgical survival
RFS	Relapse free survival
EMT	Epithelial-mesenchymal transition
F-actin	Filamentous actin
TNBC	Triple negative breast cancer
TFs	Transcription factors
OXPHOS	Oxidative phosphorylation
miRNAs	MicroRNAs
NSCLC	Non-small-cell lung cancer
