# Supplementary material for: Adipocyte-Derived Leptin Promotes PAI-1-Mediated Breast Cancer Metastasis in a STAT3/miR-34a Dependent Manner
Source: Cancers (Basel). 2020 Dec 21;12(12):3864. doi: 10.3390/cancers12123864 (PMC7767398; doi:10.3390/cancers12123864)
Supplement: Supplementary file 1 [file cancers-12-03864-s001.pdf]

cancers

MDPI

Supplementary Figures

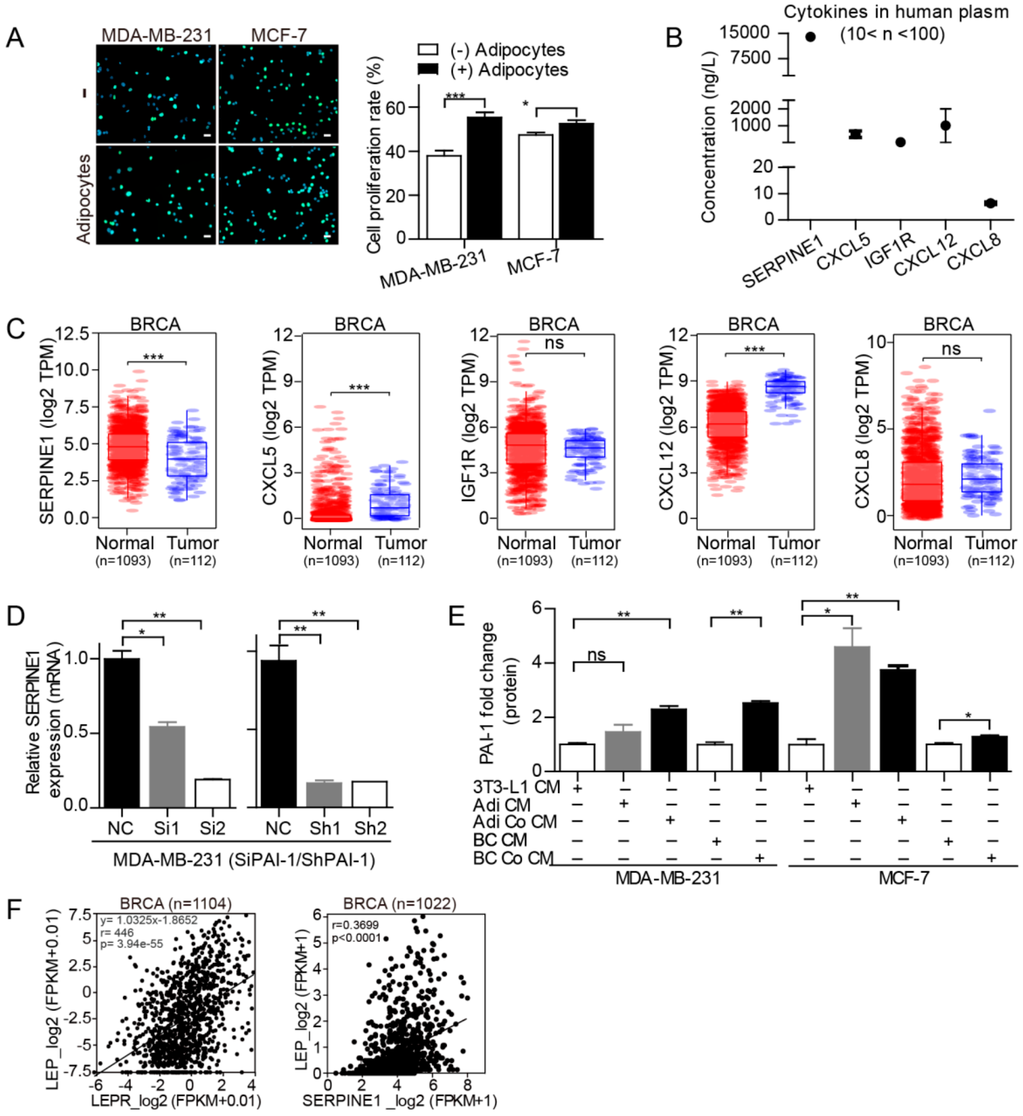

Figure S1. Edu staining and quantitative results. (A) EdU staining. Bar are means ± SEMs. (B) The concentration of cytokines in human plasm. The data is obtained from THE HUMAN PROTEIN ATLAS database (https://www.proteinatlas.org/). (C)Differential analysis of the indicated cytokines in breast cancer tissues compared with normal tissues by TCGA dataset (http://cancergenome.nih.gov/). (D) qRT-PCR. Knockdown efficiency of PAI-1 specific siRNAs and shRNAs in MDA-MB-231 cells (n=3 independent experiments). (E) Quantitative results of PAI-1 expression at the protein level in the indicated groups. (F) Correlation analysis of leptin (LEP) and leptin receptor (LEPR), as well as LEP and SERPINE1 in breast cancer (BRCA) by TCGA dataset (http://cancergenome.nih.gov/). Bars represent the means ± SEMs from three independent experiments. *p< 0.05, **p < 0.01, ***p < 0.001, ns: no significance.

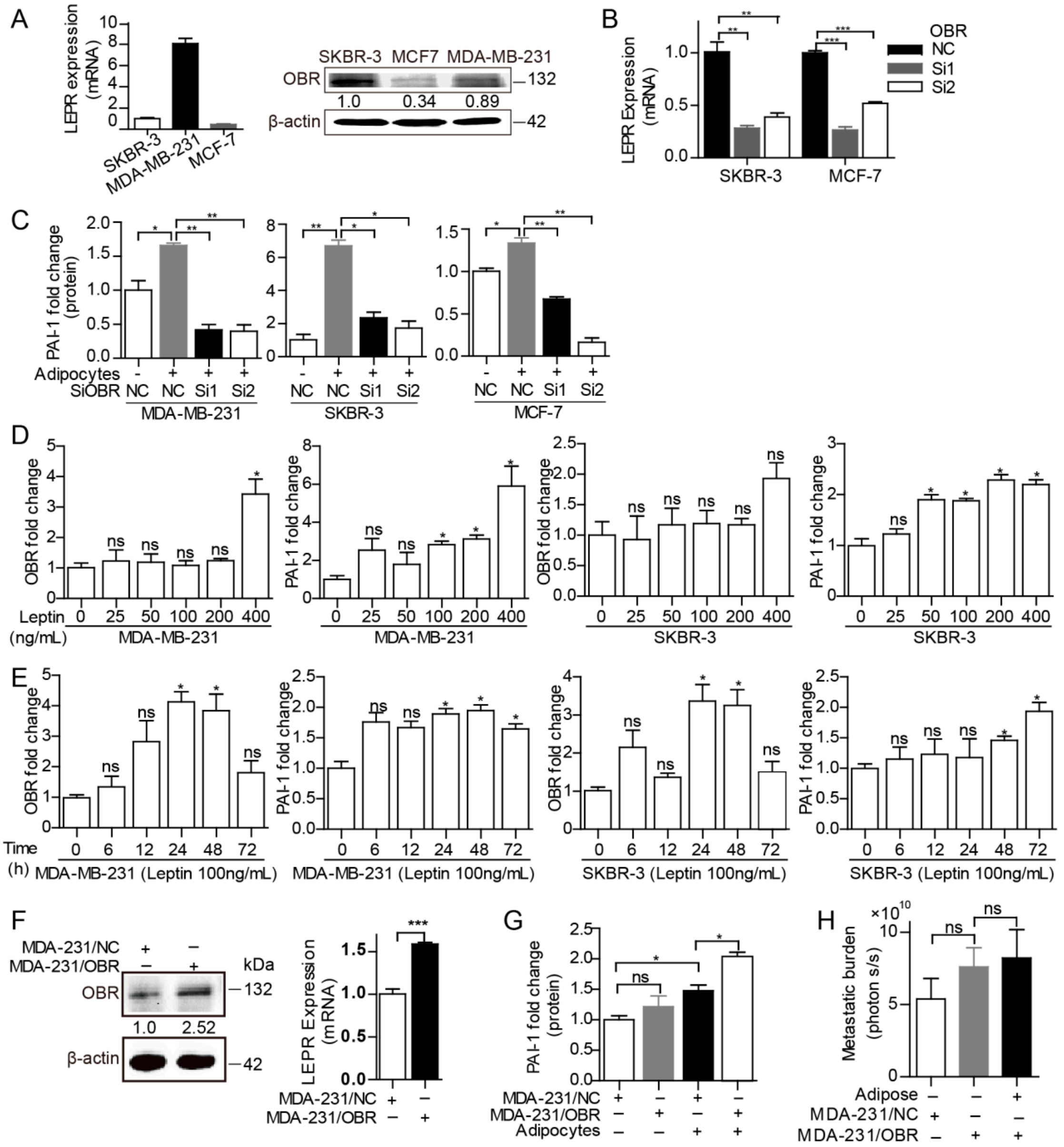

Figure S2. Quantitative results and loss and gain efficiency of OBR. (A) The expression level of OBR in breast cancer cell lines at the mRNA and protein levels. (B) Knockdown efficiency of OBR by the OBR-specific siRNAs. (C) Quantitative results of PAI-1 protein expression in the indicated groups. (D-E) Quantitative results of OBR and PAI-1 protein levels in the indicated groups. (F) The overexpression efficiency of OBR in MDA-MB-231 cells. (G) Quantitative results of PAI-1 protein expression in the indicated groups. (H) Quantitative results of the bioluminescence in Fig.6H. Bars represent the means ± SEMs from three independent experiments. *$p < 0.05$, **$p < 0.01$, ***$p < 0.001$, ns: no significance.

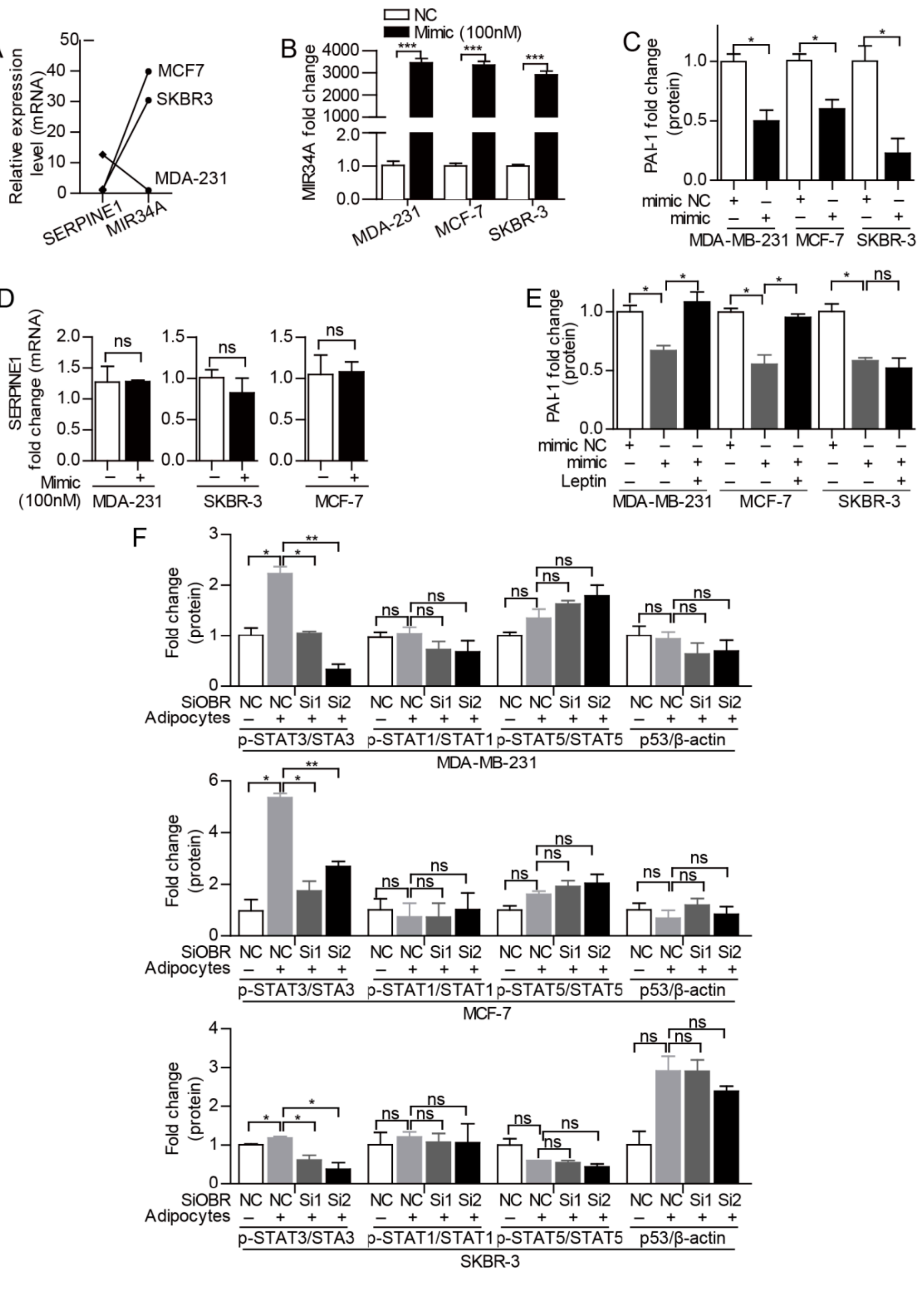

Figure S3. Quantitative results and transfection efficiency of miR-34a mimic. (A) Expression of PAI-1 and miR-34a was analyzed in different cancer cells by qRT-PCR. (B) After transfected with miR-34a mimic (100nM) for 48h, qRT-PCR was employed to estimate miR-34a expression in cancer cells. (C) Quantitative results of PAI-1 protein levels in the indicated groups. (D) Breast cancer cells were transfected with miR-34a mimic (100nM, 48h) followed by analysis of PAI-1 expression at mRNA level by qRT-PCR. (E) Quantitative results of PAI-1 protein levels in the indicated groups. (F) Quantitative results of the indicated proteins in the indicated groups. Bars represent the means ± SEMs from three independent experiments. $^{*}p < 0.05$, $^{**}p < 0.01$, $^{***}p < 0.001$, ns: no significance.

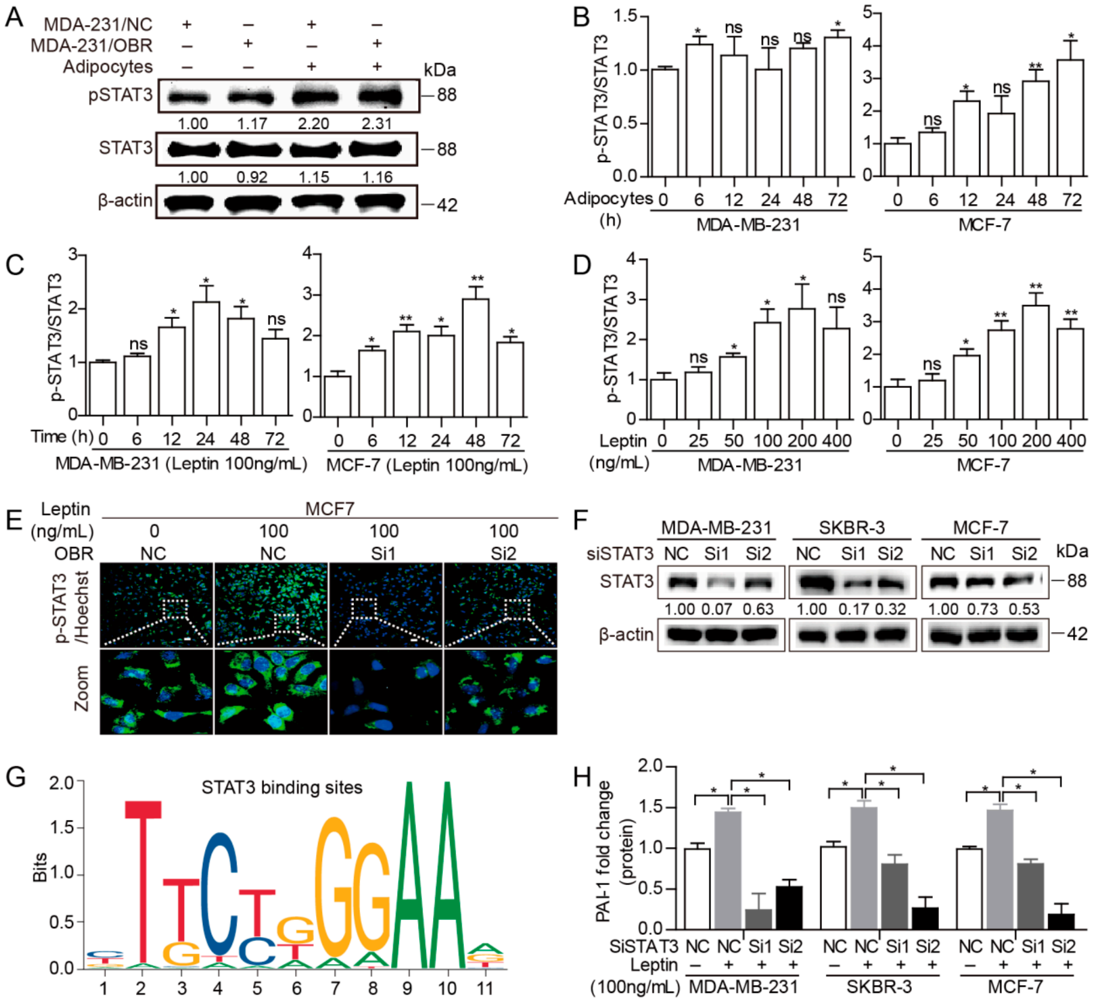

Figure S4. Quantitative results and silence efficiency of STAT3. (A) MDA-MB-231 cells with OBR ectopic expression were cultured ± adipocytes for 72h, and then western blot was carried out for the indicated proteins. (B-D) Quantitative results of the indicated proteins in different groups. (E) Immunofluorescence for p-STAT3 in MCF-7 cells after transfected with OBR siRNA or NC followed by leptin treatment (100ng/mL, 72h). Scale bar=100 μm. (F) Validation of STAT3 knock down efficiency in breast cancer cells with STAT3 specific siRNA stransfected for 72h. (G) Conservative STAT3 binding sites through the analysis of JARSPAR database. (H) Quantitative results of PAI-1 protein levels in the indicated groups. Bars represent the means ± SEMs from three independent experiments. *$p < 0.05$, **$p < 0.01$, ***$p < 0.001$, ns: no significance.

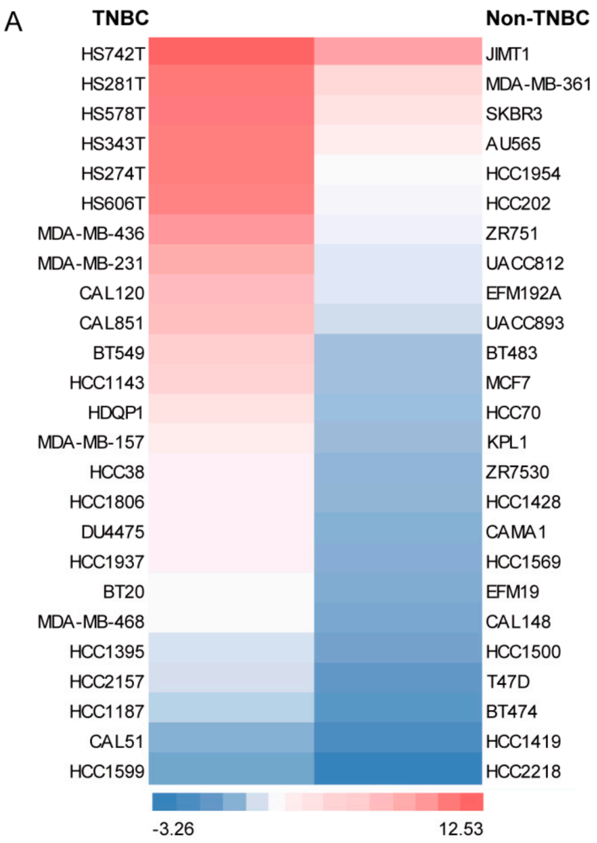

Figure S5. Expression of PAI-1 in different breast cancer cell lines. (A) Higher PAI-1 expression was detected in most TNBC cells lines compared with non-TNBC cells. The data was obtained from Cancer Cell Line Encyclopedia (RNA-seq, https://portals.broadinstitute.org/ccle).
